# Inflammation-Related Patterns in the Clinical Staging and Severity Assessment of Chronic Kidney Disease

**DOI:** 10.1155/2019/1814304

**Published:** 2019-10-07

**Authors:** Simona Mihai, Elena Codrici, Ionela D. Popescu, Ana-Maria Enciu, Elena Rusu, Diana Zilisteanu, Laura G. Necula, Gabriela Anton, Cristiana Tanase

**Affiliations:** ^1^Biochemistry-Proteomics Department, Victor Babes National Institute of Pathology, Splaiul Independentei 99-101, 050096 Sector 5, Bucharest, Romania; ^2^Cellular and Molecular Medicine Department, Carol Davila University of Medicine and Pharmacy, No. 8 B-dul Eroilor Sanitari, 050474 Sector 5, Bucharest, Romania; ^3^Fundeni Clinic of Nephrology, Carol Davila University of Medicine and Pharmacy, Sos Fundeni 258, 022328 Sector 2, Bucharest, Romania; ^4^Nephrology Department, Fundeni Clinical Institute, Sos Fundeni 258, 022328 Sector 2, Bucharest, Romania; ^5^Molecular Virology Department, Stefan S. Nicolau Institute of Virology, Sos Mihai Bravu 285, 030304 Sector 3, Bucharest, Romania; ^6^Titu Maiorescu University, Cajal Institute, Faculty of Medicine, Strada Dâmbovnicului 22, 040441, Sector 4, Bucharest, Romania

## Abstract

Chronic kidney disease (CKD) is an irreversible loss of kidney function, and it represents a major global public health burden due to both its prevalence and its continuously increasing incidence. Mineral bone disorders (MBDs) constitute a hallmark of CKD, and alongside cardiovascular complications, they underlie a poor prognosis for these patients. Thus, our study focused on novel CKD biomarker patterns and their impact on the clinical staging of the disease. As a first testing approach, the relative expression levels of 105 proteins were assessed by the Proteome Profiler Cytokine Array Kit for pooled CKD stage 2–4 serum samples to establish an overall view regarding the proteins involved in CKD pathogenesis. Among the molecules that displayed significant dysregulation in the CKD stages, we further explored the involvement of Dickkopf-related protein 1 (Dkk-1), a recognised inhibitor of the Wnt signalling pathway, and its crosstalk with 1,25OH_2_D_3_ (calcitriol) as new players in renal bone and vascular disease. The serum levels of these two molecules were quantified by an ELISA (76 samples), and the results reveal decreasing circulating levels of Dkk-1 and calcitriol in advanced CKD stages, with their circulating expression showing a downward trend as the CKD develops. In the next step, we analysed the inflammation and MBD biomarkers' expression in CKD (by xMAP array). Our results show that the molecules involved in orchestrating the inflammatory response, interleukin-6 (IL-6) and tumour necrosis factor alpha (TNF*α*), as well as the mineral biomarkers osteoprotegerin (OPG), osteocalcin (OC), osteopontin (OPN), and fibroblast growth factor 23 (FGF-23), correlate with Dkk-1 and calcitriol, raising the possibility of them being potential useful CKD biomarkers. These results reveal the impact of different biomarker patterns in CKD staging and severity, thus opening up novel approaches to be explored in CKD clinical management.

## 1. Introduction

Chronic kidney disease (CKD) represents a major global disease that covers all degrees of injured renal function, with a rising incidence and prevalence of kidney failure resulting in poor outcomes and high economic costs. According to the Kidney Disease Improving Global Outcomes (KDIGO) 2017 Clinical Practice Guideline Update for the Diagnosis, Evaluation, Prevention, and Treatment of Chronic Kidney Disease: Mineral and Bone Disorder (CKD-MBD), the disease is defined as “abnormalities of the kidney structure or function, present for more than 3 months, with implications for health” [1]. The characteristic features of CKD are the progressive and irreversible loss of renal function, which results in extensive kidney damage, leading unconditionally to end-stage renal disease (ESRD). Over the last 10 years, CKD has reached epidemic proportions, with a constant increase in terms of both prevalence and incidence, and it has been classified by the Global Burden of Disease Study as “the 12th most common cause of death, accounting for 1.1 million deaths worldwide.” Overall, its poor prognoses ranked CKD as “the 17th leading cause of global year loss of life and the 3rd largest increase of any major cause of death” [2, 3].

Cardiovascular disease (CVD) is noted as the main cause of morbidity and mortality in these patients, while CKD is considered an accelerator of cardiovascular events and an independent risk factor for CVD. It was also shown that all CKD stages are accompanied by an elevated risk of cardiovascular complications and a decreased quality of life [4].

The causes of high cardiovascular mortality related to CKD have been attributed in part to CKD-MBD syndrome, which generates a unique environment that accelerates vascular calcification (VC)—the pathological deposition of calcium phosphate in the vasculature's medial layer. Even in the early CKD stages, the systemic mineral metabolism and bone composition begin to alter; thus, the dysregulation of mineral metabolism is considered a key player in CKD pathophysiology.

An imbalance in the kidney-vascular-bone axis, a multifaceted active process, is induced by mineral metabolism disorders and also by local inflammation; nevertheless, the most extensive mineral disorders are experienced by patients suffering from CKD [5].

The discovery of Wnt inhibitors, among them Dickkopf-related protein 1 (Dkk-1), released during renal repair as crucial components of mineral bone disorder (MBD) pathogenesis, suggests that additional pathogenic factors need to be explored [6, 7].

Elucidating the signalling pathways involved in vascular smooth muscle cell calcification holds the promise of being able to unravel novel therapeutic approaches counteracting the progression of MBDs in CKD.

Various factors mediate the VC mechanisms including disturbances in the serum calcium/phosphate balance, systemic and local inflammation, the receptor activator of nuclear factor kappa B (RANK)/RANK ligand (RANKL)/osteoprotegerin (OPG) triad, aldosterone, microRNAs, osteogenic transdifferentiation, and the effects of vitamins [8]. The emerging role of 1,25-dihydroxyvitamin D_3_ (calcitriol, 1,25OH_2_D_3_) in CKD has been extensively explored, since vitamin D deficiency/insufficiency is known to be common among patients with CKD or in those undergoing dialysis. Vitamin D has pleiotropic effects on the immune, cardiovascular, and neurological systems, and many extrarenal organs have the enzymatic capability to convert 25OHD_3_ to 1,25OH_2_D_3_. It was also hypothesised that serum 1,25OH_2_D_3_ and 25OHD expressions tend to positively correlate, together with the renal function, as well [9].

Persistent low-grade inflammation is currently considered an essential part of CKD and as a traditional risk factor for renal pathology, hugely contributing to the development of all-cause mortality in these patients [10]. The role of proinflammatory cytokine overexpression inside the renal patient's landscape has drawn considerable attention, and various studies have explored the potential link between inflammatory status and renal function decline [11, 12]. A challenging theory regarding the direct consequence of inflammation on the progression of both CKD and CVD was developed based on the supposition of this association between markers of inflammation and an estimated glomerular filtration rate (eGFR) imbalance [13].

Despite the accessibility to the studies published in the past few years, the KDIGO Guideline Committee underlines the lack of strong clinical proof, emphasizing the critical role of understanding the mechanisms underlying the disease's development, yet stressing the need for comprehensive, accurate clinical trials in this direction [14].

Considering the aforementioned aspects, in this study, the correlation between the severity of CKD and inflammatory factors, MBD biomarkers, and other novel biomarkers with an impact on CKD's pathophysiology was investigated to reveal potential proteome patterns that better characterise the condition characteristic of each stage of CKD.

## 2. Materials and Methods

### 2.1. Patients and Samples

#### 2.1.1. Study Population

We included 56 patients in our cross-sectional study who were diagnosed with CKD according to the KDIGO Guidelines alongside 20 normal controls. The CKD patients were divided into three groups based on the CKD staging criteria as follows: 16 patients with CKD stage 4 (25% female and 75% male; mean age 63 ± 14.8), 26 with CKD stage 3 (31% female and 69% male; mean age 68 ± 8.5), and 14 with CKD stage 2 (29% female and 71% male; mean age 65 ± 10.3). Written informed consent was obtained from all subjects prior to their inclusion in the study according to the Helsinki Declaration and Ethics Committee that approved this study.

Patients with acute infections, acute heart failure and significant heart valvular disease, chronic use of glucocorticoids and immunosuppressive agents, and known malignancy were excluded from our study. In addition, in order to avoid the potential bias, patients undergoing vitamin D synthetic analogue treatment were also excluded.

#### 2.1.2. Clinical and Biochemical Assessment

On the day the blood samples were collected, clinical and anthropometric data were gathered: age, sex, weight, height, medical history, and concomitant treatment. Laboratory tests were performed on admission, namely, haemoglobin, haematocrit, serum creatinine, urea, uric acid, glucose, total cholesterol, triglycerides, alkaline phosphatase, phosphate, calcium, albumin, and fibrinogen. The eGFR was calculated based on the CKD-epidemiology collaboration (EPI) equation. Urinary protein excretion was determined from a 24 h urine sample.

The blood samples were harvested the morning after a 12 h fast. After a standard centrifugation, the serum was aliquoted and stored at −80°C pending further analysis.

### 2.2. Human Dot-Blot Proteome Profiler

Semiquantitative immunodetection of serum cytokines, chemokines, growth factors, angiogenesis markers, and other soluble proteins was performed using the immuno-dot-blot method in the Proteome Profiler Human XL Cytokine Array Kit (ARY022B, R&D Systems, Inc., Abingdon, UK). A number of 105 captured antibodies, along with reference controls, were spotted in duplicate on nitrocellulose membranes and incubated overnight with 100 mL of pooled serum samples. Each of the four pools was obtained by mixing the serum samples from CKD patients in stages 4, 3, and 2, respectively; the 4th pool was assigned to control sera. The protocol recommended by the manufacturer was followed accordingly. The membranes were incubated with biotinylated detection antibodies, streptavidin-horseradish peroxidase (HRP), and chemoluminescent detection reagents. Chemiluminescence signals, corresponding to the amount of protein bound, were detected using the MicroChemi 4.2 System (DNR Bio-Imaging Systems, Israel), and the intensity of the chemiluminescence signals (pixel densities) was measured using ImageJ 1.42 software (National Institute of Health, Bethesda, MD, USA). For each measured analyte, the average signal of the duplicate spots was determined and normalised to the average signal of the reference spots after being corrected with the background signal.

### 2.3. ELISA Immunoassay

Dkk-1 serum levels were assessed using the Quantikine ELISA Human Dkk-1 Immunoassay Kit (R&D Systems, Inc., USA) according to the manufacturer's protocol. The quantitative determination of the calcitriol (1,25OH_2_D_3_ (1,25-dihydroxyvitamin D_3_)) serum levels was made using the EIAab ELISA General Calcitriol Kit (Wuhan EIAab Science Co., Ltd., China), and the manufacturer's instructions were followed accordingly. The optical densities were measured using an Anthos Zenyth 3100 Microplate Multimode Detector.

### 2.4. Luminex xMAP Array Analysis

The Luminex xMAP array procedure was performed according to the manufacturer's instructions. The serum levels of the 6-plex analytes were simultaneously quantified on the Luminex 200 multiplexing platform. The Luminex xMAP array technique is based on proprietary colour-coded microspheres coated with specific capture antibodies. After the analytes from the serum samples were captured by the bead cocktail, a biotinylated detection antibody was added. The reaction mixture was then incubated with the reporter molecule conjugate (streptavidin-phycoerythrin (SA-PE)) to complete the reaction on the surface of the microspheres. After the reaction steps had been completed, the microspheres were passed rapidly through a red laser which excited the internal dyes, thus identifying each unique microsphere set. The green laser excited PE, the fluorescent dye on the reporter molecule, which was directly correlated with the amount of analyte found in the sample. All the acquired data was processed by high-speed digital-signal processors and by xPONENT 3.1 software, generating results expressed in pg/mL.

Cytokine levels and BMD biomarkers were assayed using the MILLIPLEX MAP Human Bone Magnetic Bead Panel Kit (Merck-Millipore, Billerica, MA, USA), which comprises a cocktail of six analytes: proinflammatory cytokines IL-6 and TNF-*α* and the MBD biomarkers OPG, osteocalcin (OCN), osteopontin (OPN), and fibroblast growth factor 23 (FGF-23).

For all the biological specimens, duplicate samples were used and their average concentrations were taken into consideration for further statistical analysis.

### 2.5. Statistical Analysis

As a first statistical approach, we applied the Kolmogorov-Smirnov and D'Agostino and Pearson normality tests to all the CKD and control samples under analysis. The Kolmogorov-Smirnov test was used to evaluate the normality of the data distribution. The groups presented with a nonnormal distribution (*p* < 0.0001); therefore, nonparametric statistical tests were used for further analysis. The groups were not homogeneous in terms of age and gender, but according to the results obtained after applying the Chi-square test, they did not influence the level of the analysed molecules; age was expressed as the mean ± SD. The differences between the variables were analysed using the Kruskal-Wallis test (a one-way analysis of variance) followed by a Bonferroni post hoc test to compare the results inside the different CKD stage groups. The Chi-square test for trends was applied to reveal the differences in molecule expression between the various CKD stages. The differences between the nominal variables were analysed using Chi-square tests (*r*, *p*). A value of *p* < 0.05 was considered statistically significant (^∗^*p* < 0.05, ^∗∗^*p* < 0.01, and ^∗∗∗^*p* < 0.001). Spearman's correlation analysis was used to evaluate the correlations between the analysed markers (*r*, *p*). GraphPad Prism version 5 software for Windows was used for the statistical analysis.

## 3. Results and Discussion

### 3.1. Proteome Profiler for CKD Clinical Staging by Dot-Blot Array Assessment

**Figure 1:**
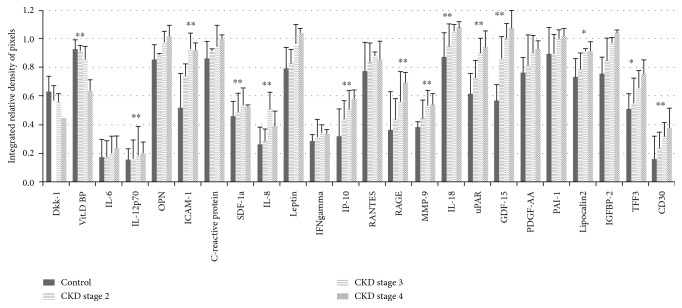
Original dot-blot membranes of the Proteome Profiler corresponding to different CKD stages and the control. The representative molecules that exhibited significant fold changes versus the control and were the subject of further analysis have been marked accordingly.

An overall perspective on the multiple proteins that are differentially expressed in the CKD stages and thus potentially influence CKD's pathophysiology was gained by performing semiquantitative dot-blot immunodetection [15, 16]. Out of 105 molecules included in the Proteome Profiler Human XL Cytokine Array Kit, 24 relevant molecules were identified as expressing significant levels in CKD patients versus the control group. At first glance, the dot-blot analysis revealed that molecules orchestrating the inflammatory response were significantly overexpressed in CKD; moreover, the multianalyte screening showed different patterns of expression depending on the CKD stage (as illustrated in Figure 1). The integrated relative pixel density of these molecules trended towards a progressive pattern of expression, exhibiting gradual amounts depending on the stage of renal disease. The most significant expression level for proteins was identified in CKD stage 4. Among the proteins that displayed a significant fold change versus the control (about a 1.5-fold change), markers for inflammatory response were identified, reflecting the high significance of the inflammatory component in CKD. Among them, IL-6, IL-8, IL-12, IL-18, interferon gamma (IFN-*γ*), the regulated upon activation normal T-cell expressed and secreted (RANTES), the receptor for advanced glycation end products (RAGE), intercellular adhesion molecule 1 (ICAM-1), inducible protein 10 (IP-10), plasminogen activator inhibitor 1 (PAI-1), platelet-derived growth factor (PDGF), and others were identified as having a place in the CKD proteome pattern, as shown in Figure 1.

Persistent, low-grade inflammation constitutes a common feature of the disease, which accompanies CKD from its onset [17]. Inflammatory biomarkers such as C-reactive protein and IL-6 are known to independently predict mortality in these patients. The origins of inflammation in kidney disease are multifactorial, including the imbalance between proinflammatory increased production, induced on the one hand by various sources of inflammatory stimuli (oxidative stress, acidosis, comorbidities, genetic and epigenetic influences, etc.) and on the other hand by their insufficient elimination due to impaired glomerular filtration [18]. IL-6 hastens the development of CKD not only by aggravating kidney injury but also by initiating its complications, especially the cardiovascular ones. It is well established that IL-6 initiates the endothelial injury mostly by reducing endothelial nitric oxide synthase and adiponectin (an antiatherogenic adipokine) expression, thus contributing to the increased incidence of cardiovascular events in CKD patients. Taken together, an increased IL-6 level not only is a consequence of CKD but also acts as a trigger for CKD-related complications [19].

Mediators of inflammation have been shown to be at high levels in CKD patients. IL-12 and IL-18 are elevated during the earlier stages of CKD, and the association with eGFR suggests that IL-18 is mainly dependent upon renal clearance, as suggested by Yong et al. [20].

The urokinase receptor system, a key regulator at the intersection between inflammation, immunity, and coagulation [21], has also been shown to significantly increase in CKD patients. Nuclear factor kappa B (NF-*κ*B), a pivotal mediator of inflammatory responses through triggering the prototypical proinflammatory signalling pathway, appears to mediate renal inflammation in different cell types including renal cells, innate immune cells, and lymphocytes [22, 23]. It was shown that NF-*κ*B also controls several genes involved in inflammation, and RAGE (an advanced glycation end-product-specific receptor) itself seems to be upregulated by NF-*κ*B [24].

The pleiotropic cytokine OPN is increased in early CKD stages, and its circulatory level increases with the severity of the disease stage. OPN is an important factor in bone remodelling, as it is involved in the pathogenesis of both kidney and cardiovascular diseases. Barreto et al. reported a positive correlation between OPN levels and the clinical outcomes of CKD patients depending on their inflammatory status [25].

**Figure 2:**
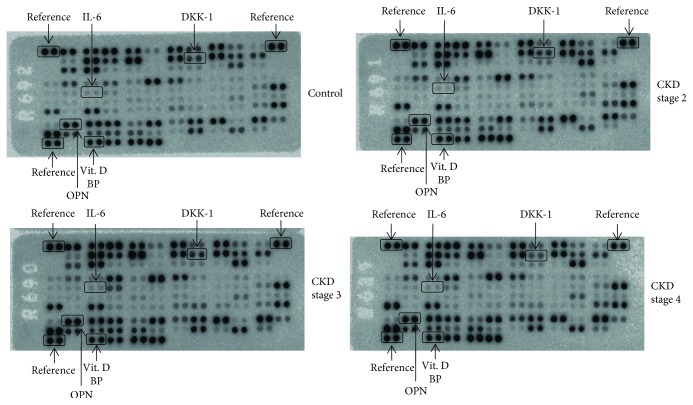
Serum protein profiling in CKD stages 2–4 versus the control. The integrated relative density of the pixels was calculated for each molecule after normalisation to the average signal of the reference spots. The molecules showed an ascending trend of expression according to the severity of the disease; Dkk-1 and vit D BP showed a descending trend.

**Figure 3:**
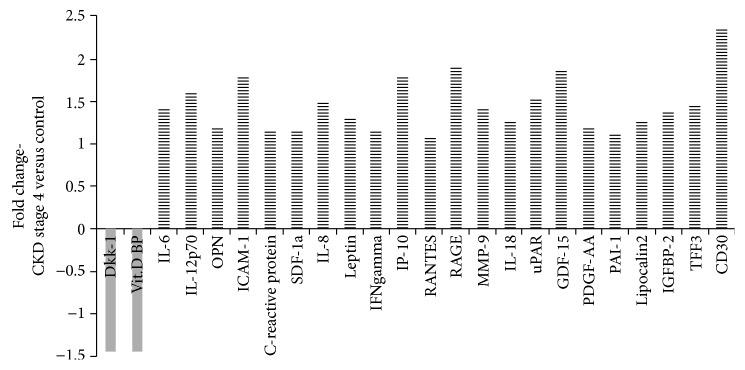
The fold change in protein expression in CKD stage 4 versus the control. The average for the control group was established at 1.0, and for each analysed molecule, the fold change was expressed as the CKD stage 4/control ratio.

The interplay between different proteins involved in inflammation and the MBD profile is depicted in Figures 1 and 2. Among the molecules that exhibited significant downregulation in CKD stage 4 versus the control (with about a 1.5-fold change, *p* < 0.05—illustrated in Figure 3), Dkk-1 and vitamin D binding protein (vit D BP) showed the highest potential and were chosen for further analyses. The proinflammatory cytokine IL-6 and the MBD biomarker OPN, with significant increases in CKD, were also subject to further analyses.

### 3.2. Dkk-1 Was Negatively Correlated with CKD Clinical Staging

Recent studies emphasize the close connection between CKD and cardiovascular complications, as well as the presence of a dysregulated Wnt signalling pathway in CVD.

[26, 27]. Based on these facts, we explored the circulating expression of Dkk-1, a recognised inhibitor of the Wnt–*β*-catenin signalling pathway, in modulating the renal disease course. Targeting the Wnt signalling cascade aligns with innovative therapeutic CVD strategies [28, 29].

**Figure 4:**
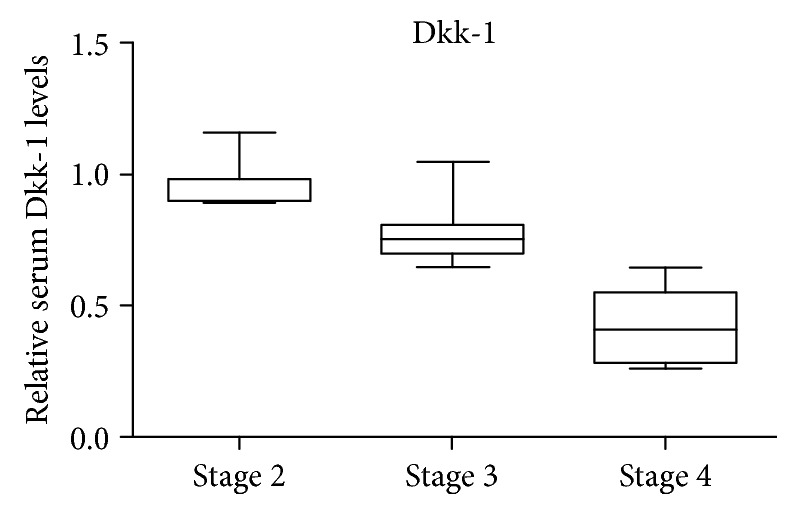
Dkk-1 fold change expression in CKD stages 4, 3, and 2 versus the control, assessed by ELISA.

The significant downregulation of Dkk-1 in CKD stage 4, determined via a dot-blot analysis, was further confirmed by running a quantitative ELISA. Our results showed a statistically significant decreased expression of Dkk-1 in CKD patients compared to the control group (*p* < 0.05, Figure 4).

Relative serum Dkk-1 levels decreased even in the early stages of CKD, with a 1.05-fold decrease in stage 2 versus the control and a 1.3-fold decrease in stage 3. Dkk-1 circulating levels showed a downward trend, culminating in stage 4, where a significant 2.36-fold decrease was recorded versus the control. Recent studies have also reported that serum Dkk-1 levels were lower in CKD patients as compared with controls and that Dkk-1 levels had a tendency to decrease with the progressive development of CKD [30]. Interestingly, Behets et al. reported lower levels in CKD patients than in the controls, but Dkk-1 levels were not associated with the laboratory parameters of mineral metabolism. Since these correlations were applied only to haemodialysis patients, it was hypothesised that Dkk-1 targeted different regulatory mechanisms inside the Wnt–*β*-catenin signalling pathway [31]. Thus, Dkk-1 seems to have distinct effects depending on the cell type, which is in line with the different effects of Wnt–*β*-catenin signalling. Increasing evidence indicates that the Wnt–*β*-catenin signalling pathway has important roles in skeletal development and bone mass equilibrium. It was found that Wnt activation increases bone formation and reduces bone desorption; therefore, a disturbed Wnt–*β*-catenin signalling pathway may be involved in CKD-MBD pathophysiology [32, 33]. Since VC is a hallmark feature of chronic inflammatory disorders, it has been shown that CKD aggravates vascular inflammation [34]. In a study conducted by Jang et al., the role of Dkk-1 in mediating the inflammatory response was investigated, thus exploring the implications of the Wnt signalling pathway in promoting immune responses or inflammation by triggering NF-*κ*B activity. In this study, lipopolysaccharide- (LPS-) induced inflammatory responses were found to be prevented by Dkk-1 in a dose-dependent manner in human bronchial epithelial cells and human umbilical vein endothelial cells (HUVEC). Therefore, LPS-induced expression of the proinflammatory cytokines IL-6 and IL-8 was inhibited by Dkk-1. Other proinflammatory genes such as TNF-*α* and IL-1*β* were also downregulated by Dkk-1, a secreted Wnt antagonist [35].

Exploring the potential of the Wnt–*β*-catenin signalling pathway inhibitor Dkk-1 in predicting the severity of CKD and elucidating its role in CKD-MBD pathophysiology is thus a promising strategy for further studies.

### 3.3. Calcitriol Levels Decrease with Increasing CKD Stage

A vitamin D deficiency is a common condition associated with kidney disease. Many clinical studies have highlighted how a vitamin D deficiency is an important risk factor for CKD patients [36, 37].

Since dot-blot screening revealed significantly decreased levels of vitamin D BP, we thus measured the most active vitamin D metabolite in the kidneys: calcitriol (1,25-dihydroxyvitamin D_3_ (1,25OH_2_D_3_)).

Experimental studies have established that calcitriol and vitamin D receptors are decisive regulators of the heart in terms of structure and function. In addition, clinical studies have correlated vitamin D deficiency with CVD. Emerging evidence has highlighted that calcitriol is significantly involved in CVD-related signalling pathways, particularly in the Wnt signalling pathway [38].

**Figure 5:**
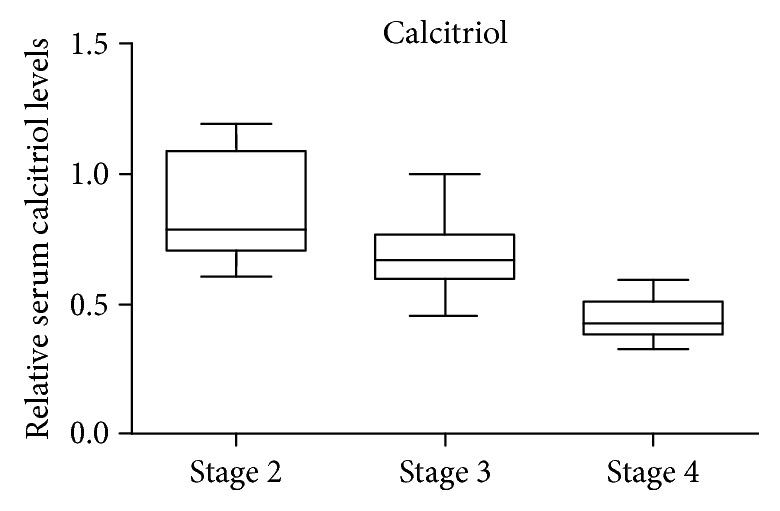
Calcitriol fold change expression in CKD stages 4, 3, and 2 versus the control, assessed by ELISA.

Our results revealed that relative serum calcitriol levels started to decrease even in the early CKD stages, showing a 1.15-fold decrease in stage 2 compared to the control condition, a 1.5-fold decrease in stage 3, and a 2.24-fold decrease in stage 4, respectively (as depicted in Figure 5), thus gradually decreasing as the disease develops. In the early CKD stages, the physiologic FGF-23 secretion from the osteocytes causes inhibition of 1-*α*-hydroxylase and stimulation of 24-hydroxylase in proximal renal tubules, thereby decreasing calcitriol production. As CKD evolves, the decrease in the functioning nephron mass combined with hyperphosphatemia and high FGF-23 levels also results in calcitriol deficiency [6, 39]. Since inflammation has emerged to be at the core of CKD pathophysiology, it was also hypothesised that vitamin D has a potential role in modulating inflammatory cytokines and oxidative stress, but the molecular mechanisms still remain unclear [40]. Recent studies have shown that vitamin D supplementation among CKD patients undergoing dialysis had beneficial effects on several genes related to inflammation and oxidative stress. The downward trend in calcitriol concentrations in the CKD groups could be related to various inflammatory and MBD factors, thus providing the basis for future clinical assessments.

### 3.4. Multiplexing Showed Inflammatory and Mineral Bone Disorder Biomarker Levels to Be Positively Correlated with Disease Severity

**Figure 6:**
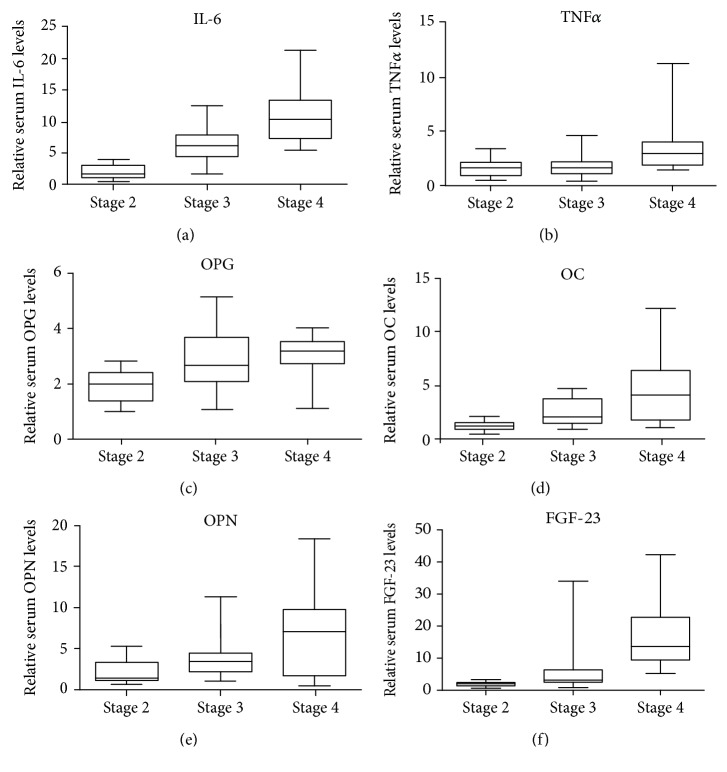
Fold change in serum IL-6 (a), TNF*α* (b), OPG (c), OC (d), OPN (e), and FGF-23 (f) expressions in CKD stages 4, 3, and 2 versus the control, assessed by xMAP array.

Among the many contributors to CKD's poor prognosis, systemic low-grade inflammation is one of the major players with an impact on the uremic phenotype in CKD. This chronic condition is fuelled by several independent mechanisms, among which the mediators of inflammation, IL-6 and TNF*α*, play important roles. We have simultaneously quantified the serum levels of IL-6 and TNF*α* using the Luminex multiplexing xMAP array platform, assaying via a preconfigured cytokine kit. Our results revealed that relative serum IL-6 levels started to increase in CKD early stage 2, showing a 1.9-fold change compared to the control, with an ascending trend, presenting with a 6.3-fold increase in stage 3 and an 11-fold increase in stage 4 (Figure 6(a)). Analysing the circulating expression of TNF*α*, we also observed an ascending trend, with a 3.3-fold increase in stage 4 versus the control, and as for CKD stages 3 and 4, the increases were 1.8-fold and 1.7-fold, respectively (Figure 6(b)).

Given the fact that various cytokines mediate the inflammatory response, the extent to which inflammation plays a role in raising the risk of MBDs in CKD remains unclear. Regarding the MBD molecules, we analysed the serum levels for OPG, OC, OPN, and FGF-23. All these biomarkers presented with an upward trend of expression, correlated with disease severity. In CKD stage 4, the circulatory levels showed the most significant differences compared to the control, as follows: for OPG, a 3.14-fold increase; for OC, a 4.6-fold increase; for OPN, a 7-fold increase; and for FGF-23, a 17-fold increase. Our results suggest that the serum levels of the above-mentioned molecules start to increase progressively, even from the CKD early stage 2, as depicted in Figures 6(c)–6(f).

Since all the analysed biomarkers expressed the highest concentrations in the most advanced stage of the disease, and given that the circulatory trend increases as the disease evolves, we considered it necessary to further analyse the possible correlations between these molecules that had a potential impact on CKD pathogenesis.

### 3.5. Correlations between Orchestrators of Inflammatory Response and Biomarkers of Mineral and Bone Disorders in CKD

#### 3.5.1. The Trend for Biomarker Expression Was Modified Depending on the CKD Stage

The pathophysiologic interplay between mediators of inflammation and the molecules involved in MBDs was further analysed to establish potential significant correlations at each stage of renal disease. By applying the Chi-square test for trends, it was found that each CKD stage had its own unique biomarker signature.

In CKD stage 4, we found a strong positive correlation between Dkk-1 and calcitriol and a negative correlation between Dkk-1 and IL-6, OPG, OC, OPN, and FGF-23 (*p* < 0.001, Chi-square test for trends). Renal function (eGFR) was positively correlated with Dkk-1 in CKD stage 4 (*p* < 0.001).

Yeremenko et al. also observed an inverse correlation between Dkk-1 and IL-6 in a study on inflamed arthritic joints, potentially reflected by the differential regulation of Dkk-1 production by TNF*α* and IL-6 [41]. Besides, it was suggested that there were other recognised signalling pathways that Dkk-1 utilises other than the well-known canonical Wnt pathway [42]. Another study highlighted that the production of proinflammatory cytokines IL-4 and IL-10 was notably reduced by Dkk-1 inhibitor treatment, suggesting that Dkk-1 utilises the MAPK and mTOR signalling pathway components to induce type 2 cell-mediated immune responses or inflammation [43]. In a study conducted by Malysheva et al., it was shown that proinflammatory cytokine IL-6 repressed the activation of the Wnt signalling pathway in human synoviocyte cells, and together with TNF*α* and Dkk-1, it inhibited the activation of the Wnt response [44].

It was also found that calcitriol distinctly regulated two genes encoding the extracellular Wnt inhibitors Dkk-1 and Dkk-4 via an indirect transcriptional mechanism. Thus, calcitriol increases the expression of Dkk-1 RNA and protein, acting as a tumour suppressor in human colon cancer cells harbouring endogenous mutations in the Wnt–*β*-catenin pathway [45].

Moreover, in CKD stage 4, the serum calcitriol concentrations were significantly correlated with proinflammatory cytokine TNF*α* (*p* < 0.01, Chi-square test) and the MBD markers OC, OPN, and FGF-23.

Our findings support the hypothesis that Dkk-1 could be a useful biomarker for CKD severity, together with calcitriol, both expressing the lowest levels in CKD stage 4.

According to recent studies, serum Dkk-1 levels were lower in CKD patients, displaying different kinetics depending on the disease stage [31].

We also obtained significant correlations between Dkk-1 and calcitriol in CKD stages 3 and 2 and with several proinflammatory and MBD markers, as follows: Dkk-1 and OPG, OPN, and FGF-23 (*p* < 0.001, Chi-square test for trends) in CKD stages 3 and 2; calcitriol and TNF*α* (*p* < 0.01) in CKD stage 3; and Dkk-1 and OPG and FGF-23 (*p* < 0.001) in CKD stage 2.

According to our results, Dkk-1, calcitriol, mediators of inflammation, and MBD markers showed significant interactions, also being correlated with the severity of CKD. How the relative balance between Dkk-1 and other cytokines determines Wnt signalling and the pattern of inflammation in CKD's different stages needs to be further investigated.

#### 3.5.2. Strong Correlations between Dkk-1 and Calcitriol, Inflammatory Cytokines, and Renal Function in the CKD Patient Groups

The investigation of correlations in the CKD patient groups was examined by applying the *χ*^2^ test (*χ*^2^, *p*) for serum levels of all the above-mentioned markers, and strong correlations were found between Dkk-1 and calcitriol (*χ*^2^ = 21.4, *p* < 0.001). Furthermore, Dkk-1 was also strongly correlated with the mediators of inflammation IL-6 (*χ*^2^ = 13.7, *p* < 0.001) and TNF*α* (*χ*^2^ = 10.4, *p* = 0.001) and with the MBD biomarker FGF-23 (*χ*^2^ = 10, *p* = 0.001).

Calcitriol expression in the CKD patient groups was correlated with IL-6 (*χ*^2^ = 4.4, *p* < 0.05) and FGF-23 (*χ*^2^ = 5.5, *p* = 0.01). Regarding renal function, we found a strong correlation between eGFR and Dkk-1 (*χ*^2^ = 8.48, *p* < 0.01) and calcitriol (*χ*^2^ = 8.36, *p* < 0.01), indicating the increased potential for these two molecules in terms of assessing the severity of the disease.

In order to reveal the significant biomarker correlations between the CKD stages, we performed Spearman correlation tests (*r*, *p* value). In advanced CKD stage 4, we obtained significant correlations, as follows: TNF*α* and Dkk-1 (*r* = 0.50, *p* < 0.05), OPG (*r* = 0.58, *p* < 0.05), and OPN (*r* = 0.66, *p* = 0.001). The MBD biomarkers OPG and OPN were also correlated (*r* = 0.51, *p* < 0.05).

Other studies also supported the interactions between the key players of bone metabolism, Dkk-1 and OPG, in modulating the Wnt signalling pathway by balancing out bone absorption and reconstruction. TNF-*α*, a key inducer of Dkk-1, alongside OPG emerged as independent predictors of osteoarthritis severity. TNF-*α*, Dkk-1, and OPG were considered as valuable biomarkers in predicting the severity of the disease. The study also supported inflammation-induced Dkk-1 and OPG in osteoarthritis pathogenesis [46].

In CKD stage 3, correlations between the proinflammatory biomarkers TNF*α* and OPG (*r* = 0.6, *p* = 0.001) and FGF-23 (*r* = 0.57, *p* < 0.01) are highlighted. In CKD early stage 2, we found a strong negative correlation between Dkk-1 and FGF-23 (*r* = −0.84, *p* < 0.001); moderate correlations were also observed between calcitriol and IL-6 (*r* = 0.53, *p* < 0.05), TNF*α* (*r* = 0.58, *p* < 0.05), OPG (*r* = 0.71, *p* < 0.05), and FGF-23 (*r* = 0.52, *p* < 0.05). The mediators of inflammation, IL-6 and TNF*α*, were also moderately correlated (*r* = 0.58, *p* < 0.05), and a moderate correlation was found between IL-6 and OPG (*r* = 0.61, *p* = 0.01).

In CKD, a complex network between Dkk-1, calcitriol, mediators of inflammation, and MBD markers exists, but the level at which it can affect the course of the disease remains in question.

#### 3.5.3. Significant Differences between Dkk-1, Calcitriol, Mineral Disorders, Inflammatory Markers, and Renal Function, Depending on CKD Stages

By applying the Kruskal-Wallis one-way analysis of variance, we obtained significant differences in the circulating expression of Dkk-1, calcitriol, and eGFR in CKD patients (*p* < 0.0001). The post hoc analysis showed that levels of Dkk-1, calcitriol, and eGFR were significantly different between CKD stage 4 and stage 3, CKD stages 4 and 2, and CKD stages 3 and 2, respectively (*p* < 0.0001), highlighting the potential of these two markers in evaluating the severity of the disease.

Significant differences in IL-6 were observed in CKD patients (*p* < 0.0001). Bonferroni's multiple comparison test showed that IL-6 was significantly different between CKD stage 4 and stage 3, CKD stages 4 and 2 (*p* < 0.0001), and CKD stages 3 and 2 (*p* < 0.05). TNF*α* showed a significant variance in CKD patients (*p* < 0.01), and the differences between the stages were as follows: CKD stage 4 and stage 3 and CKD stages 4 and 2 (*p* < 0.05), according to our post hoc analysis.

FGF-23 and OC presented with significant differences in the CKD group (*p* < 0.0001), and the comparisons between stages were only significant between CKD stage 4 and stage 3 and CKD stages 4 and 2 (*p* < 0.0001).

According to our results, we can conclude that a crosstalk between Dkk-1, calcitriol, mineral disorders, inflammation, and renal function is present in CKD, thus influencing CKD pathophysiology. Inflammation, the hallmark feature of chronic diseases, seems to be a common mediator for both kidney function and subsidiary MBDs. Because of its insidious nature, CKD silently evolves alongside other chronic conditions, exhibiting different biomarker patterns depending on disease severity.

### 3.6. Functional Interplay between Markers of Inflammation and Mineral Bone Disorders in CKD

**Figure 7:**
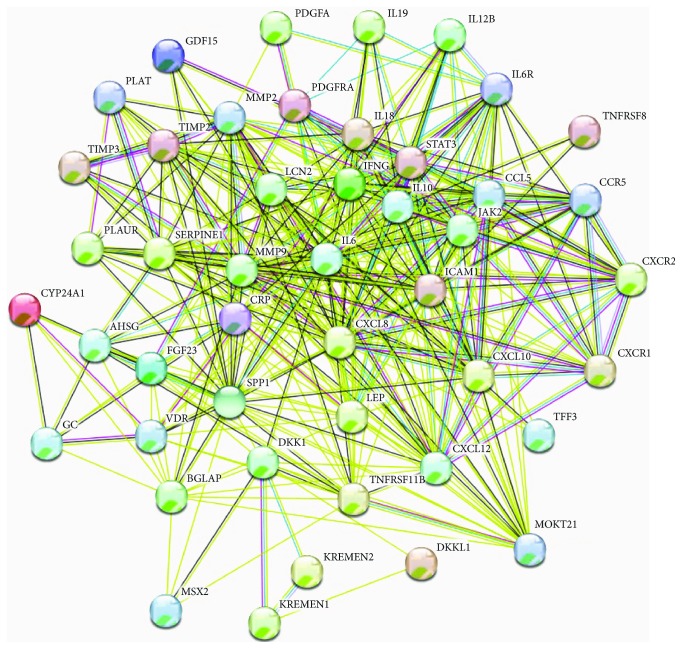
Functional interaction between different molecules involved in inflammation and MBDs in CKD. The coloured nodes are represented by query proteins and the first shell of interactors. Edges represent protein-protein functional associations, assigned with different colour codes, as follows: a blue edge indicates known interactions from curated databases, a pink edge indicates known interactions that have been experimentally determined, a green edge indicates predicted interactions in the gene neighbourhood, a red edge indicates predicted interactions for gene fusions, a blue-ink edge indicates predicted interactions for gene cooccurrences, a light-green edge indicates other interactions derived from text mining, and a black edge indicates gene coexpression derived from other databases. Abbreviations: CYP24A1: calcitriol, 1,25-dihydroxyvitamin D_3_, and 1,25OH_2_D_3_; SPP1: osteopontin, OPN; TNFRSF11B: osteoprotegerin, OPG; BGLAP: osteocalcin, OC; CXCL8: IL-8, interleukin-8; GC: vitamin D binding protein, DBP; VDR: vitamin D receptor.

Considering the relevant proteins revealed by dot-blot immunodetection screening, the functional interactions between the molecules involved in shaping the different patterns of CKD have been put together by employing the STRING databases. The interactions include functional associations between multiple molecules stemming from computational prediction, knowledge transfer between organisms, and interactions derived from other databases. Stronger evidence for an association is represented by a thicker network edge, as depicted in Figure 7.

Since CKD commonly arises alongside other comorbidities (such as hypertension, diabetes, and CVD) and the diagnosis of isolated CKD represents the exception rather than the rule, an integrative patient assessment is the best clinical approach [47]. Detailed characterisation of kidney disease is needed to better understand the molecular relationships underlying the pathophysiology of disease and to design CKD biomarker patterns characteristic of the various CKD stages, thus moving towards personalised care for each individual patient.

A potential limitation of our study is its cross-sectional design, given the relatively small number of patients included in our study. Therefore, further intense research is necessary to completely decipher the underlying mechanisms behind the connections between the analysed molecules in order to better characterise the cytokine patterns in CKD.

## 4. Conclusions

As highlighted in our study, a functional interplay occurs between markers of inflammation and MBDs in CKD depending on disease severity. In spite of the advances in CKD pathophysiology, there is an emerging need for novel biomarkers to better characterise the different patterns of nephropathy at each CKD stage. Out of all the analysed molecules, Dkk-1 and calcitriol were found to significantly correlate with CKD clinical staging, exhibiting the lowest levels in CKD stage 4. Since inflammation has emerged at the core of the pathophysiology of CKD, our results revealed significant correlations between Dkk-1 and calcitriol and proinflammatory cytokines, starting with the early CKD stages. The MBD biomarkers OPG, OPN, OC, and FGF-23 were significantly correlated with Dkk-1 and calcitriol, as well as with the mediators of inflammation IL-6 and TNF*α*. In view of these findings, Dkk-1 and calcitriol could be considered as potential useful biomarkers for CKD severity. Nevertheless, further studies are needed to clearly unravel the complex networking between Dkk-1, calcitriol, the mediators of inflammation, and MBD markers to design promising biomarker patterns for CKD, starting with its early stages.

## Data Availability

The data used to support the findings of this study are available from the corresponding author upon request.
